# Roles of sulfate-reducing bacteria in sustaining the diversity and stability of marine bacterial community

**DOI:** 10.3389/fmicb.2023.1218828

**Published:** 2023-08-10

**Authors:** Liyun An, Ying-Chun Yan, Hai-Long Tian, Chang-Qiao Chi, Yong Nie, Xiao-Lei Wu

**Affiliations:** ^1^College of Architecture and Environment, Sichuan University, Chengdu, China; ^2^College of Agriculture, Henan University, Kaifeng, China; ^3^College of Engineering, Peking University, Beijing, China; ^4^Institute of Ocean Research, Peking University, Beijing, China

**Keywords:** sediment, seawater, network analysis, assembly process, sulfate-reducing bacteria

## Abstract

Microbes play central roles in ocean food webs and global biogeochemical processes. Yet, the information available regarding the highly diverse bacterial communities in these systems is not comprehensive. Here we investigated the diversity, assembly process, and species coexistence frequency of bacterial communities in seawater and sediment across ∼600 km of the eastern Chinese marginal seas using 16S rRNA gene amplicon sequencing. Our analyses showed that compared with seawater, bacterial communities in sediment possessed higher diversity and experienced tight phylogenetic distribution. Neutral model analysis showed that the relative contribution of stochastic processes to the assembly process of bacterial communities in sediment was lower than that in seawater. Functional prediction results showed that sulfate-reducing bacteria (SRB) were enriched in the core bacterial sub-communities. The bacterial diversities of both sediment and seawater were positively associated with the relative abundance of SRB. Co-occurrence analysis showed that bacteria in seawater exhibited a more complex interaction network and closer co-occurrence relationships than those in sediment. The SRB of seawater were centrally located in the network and played an essential role in sustaining the complex network. In addition, further analysis indicated that the SRB of seawater helped maintain the high stability of the bacterial network. Overall, this study provided further comprehensive information regarding the characteristics of bacterial communities in the ocean, and provides new insights into keystone taxa and their roles in sustaining microbial diversity and stability in ocean.

## 1. Introduction

Oceans cover approximately 70% of the Earth’s surface and contain 97% of all water on our planet (Sunagawa et al., 2020). Plankton is the dominant life form in the oceans and is comprised of highly dynamic and interacting populations of bacteria, archaea, viruses, protists, and animals that drift with the currents (Barton et al., 2013; Brum et al., 2015). Together, these organisms play a significant role in the Earth’s biogeochemical system by contributing to almost half of the net primary production on the planet (Guidi et al., 2016; Carroll et al., 2022). Additionally, sediment is another essential component of the ocean ecosystem and is estimated to contain approximately half (>10^29^) of the microbial cells in the oceans (Kallmeyer et al., 2012). The global ocean sampling expedition substantially promoted the development of ocean ecosystem biology, for example the Tara Oceans project (Sunagawa et al., 2020). The sequencing data obtained from this expedition has provided unparalleled insights into the composition, diversity, function, and distribution patterns of bacterial, archaeal, and viral communities on a global scale. This knowledge has enabled us to better understand ocean biosphere.

The interactions between microbes are also crucial for maintaining a diverse microbial community. Due to their small size, high abundance, wide distribution, and short generation time, microbes interact in complex ways ranging from mutualism to competition, via exchanges of materials and energy (Faust and Raes, 2012). Such intricate ecological relationships can be represented as networks, with species as nodes and their relationships as links (Pržulj and Malod-Dognin, 2016). The development of high-throughput sequencing technologies opened a new era in microbiome studies, allowing the opportunity to systematically study the interaction between microbes from various environments using co-occurrence networks. For example, researchers have explored the influence of climate warming on grassland soil microbial network complexity based on co-occurrence networks, and found that climate warming significantly increases network complexity (Yuan et al., 2021). In soil, species coexistence within microbial communities is regulated by community assembly processes, and microbial co-occurrence associations tend to be higher when communities are primarily driven by dispersal limitation relative to species sorting (Jiao et al., 2020). In marine, researchers explored spatiotemporal dynamics of the archaeal community in coastal sediments, and found that seasonality in archaeal co-occurrence patterns, archaea were more connected in winter than in summer (Liu et al., 2020). Additionally, by linking assembly process and species co-occurrence, researchers found that microbial co-occurrence associations in the eastern Indian ocean tended to be higher when deterministic processes were weaker (Li et al., 2021).

In addition, networks are also used to investigate the dynamics of ecosystems (Montesinos-Navarro et al., 2017; Toju et al., 2017; Ullah et al., 2018). One fundamental yet hotly debated question is whether and how the complexity of ecological network affects ecosystem stability. According to Robert May’s Complexity-stability theory, large ecosystems can maintain stability up to a certain critical complexity, This means that the complexity of ecological networks constraints their stability (May, 1972). This critical complexity is determined by the number of species in the ecosystem and the intensity of interactions among them. May’s theory has been confirmed through mathematical modeling (Thébault and Fontaine, 2010; Mougi and Kondoh, 2012; Gregorini et al., 2018; Qian and Akçay, 2020). Furthermore, over the past decade, May’s theory has been examined in real-world ecosystems, where species richness is high and species interactions are complex. After understanding interaction relationships in advance through numerous cultivation experiments. Researchers meticulously constructed ecological interactions networks among species, and investigated the relationships between species diversity, interactions, and community stability. They discovered that May’s theory is not universally applicable in natural ecosystems (Yodzis, 1981; Pimm et al., 1991; de Ruiter et al., 1995). Recently, some researchers have introduced a new computational framework for estimating the complexity of ecosystems. This framework does not rely on *a priori* knowledge of the underlying interaction network. Their findings indicate that in natural communities, the relationship between complexity and stability aligns with May’s theory. Specifically, there is a pronounced trade-off between the number of species and their interactions. Natural communities maintain stability by decreasing complexity (Yonatan et al., 2022).

While it is well documented that interaction between species have a significant impact on community structure and ecosystem stability, there is limited understanding regarding the effects of SRB on the structure and stability of bacterial networks in the ocean. The aims of this study were to (I) investigate microbial variations among various habitats in eastern Chinese marginal seas; (II) gain insight into the ecological role and significance of SRB in bacterial networks; (III) assess the influence of SRB on the stability of bacterial communities in ocean ecosystems. Given the crucial contributions of SRB in ocean ecosystems, understanding the influence of SRB on the community stability can promote the overall stability and functionality of ocean ecosystems.

## 2. Materials and methods

### 2.1. Sampling and data collection

From 2011 to 2014, we collected 594 sediment samples (240–2,000 m deep) and 110 seawater samples (0–1,500 m deep) from eastern Chinese marginal seas (Supplementary Figure 1). For bacterial community analyses, 3 L seawater samples were filtered through a 0.22-μm-pore polycarbonate membrane. All membranes and sediment were immediately frozen at −20°C in the field and stored at −80°C in the lab until DNA extraction.

### 2.2. DNA extraction, PCR, and sequencing

Extraction and purification of total DNA from 0.5 g of sediment samples and 0.22 μm membranes were carried out using the Power soil DNA Isolation Kit according to the manufacturer’s protocols. Barcode sequences were ligated to the PCR primers before amplification for pooling of multiple samples within a single sequencing run. The primer set 344F (5′-CCT ACG GGA GGC AGC AG-3′) and 1073R (5′-ACG AGC TGA CGA CAR CCA TG-3′) was used for bacterial 16S rRNA gene amplification. Gene amplification was conducted in a 50 μL reaction system containing 10 μL of TransStart FastPfu Buffer (10 ×), 5 μL of dNTP mix (5 mM), 2.5 μL of each primer (5 pmol/μl), 1 μL of TransStart Fastpfu polymerase (2.5 U/μl), 2.5 μL of template DNA, and 26.5 μL of H_2_O. The PCR parameters were 95°C for 2 min, followed by 25 cycles of 95°C for 30 s, 56°C for 30 s, and 72°C for 30 s, with a final extension at 72°C for 5 min. The resulting barcoded PCR product was normalized in equimolar amounts and sequenced on a Roche GS-FLX 454 automated pyrosequencer. All raw sequence data presented in this article were deposited in the NCBI Sequence Read Archive (SRA) database under Bioproject number PRJNA766181.

### 2.3. Operational taxonomic units (OTUs) classification and functional analysis

Raw data were denoised using the “fastx_uniques” command in USEARCH. The remaining sequences were clustered in OTUs based on 97% similarity level and chimeras were filtered using “cluster_otus” command in USEARCH (Edgar, 2010). In addition, singleton and doubleton OTUs, which may represent sequencing errors, were removed for downstream analyses using “otutab_trim” command in USEARCH. After that, OTUs taxonomy was assigned against the Greengenes database (13–8 release) using the “assign_taxonomy.py” script in QIIME1 (McDonald et al., 2012). In addition, OTU tables were resampled according to a median number of sequences from each sample (1,883 for bacteria) using “otutab_norm” command in USEARCH. In addition, to infer metabolic potential from 16S rRNA sequencing data, we used the bioinformatic tool PICRUSt (Langille et al., 2013) to predict metabolic genes of microbial communities. Metabolic genes were matched to specific pathways using the Kyoto Encyclopedia of Genes and Genomes (KEGG) (Kanehisa et al., 2012) database.

### 2.4. Neutral model

To explore community assembly, we assessed the fit of the Sloan neutral community model to the distributions of bacterial taxa (Sloan et al., 2006). In this model, the parameter *R*^2^ predicts the overall fit to the neutral model. In addition, to determine whether the neutral model was only based on random sampling, we compared the fit of the neutral model with the fit of a binomial distribution model (Burns et al., 2016). The Akaike information criterion (AIC) of each model was calculated based on 1,000 bootstrap replicates in R.

### 2.5. Network analysis

To restrain the artificial associations among low-abundance bacteria that were present with very few non-zero observations, reduce the network complexity, and better detect the core microorganisms, only OTUs with a sum of relative abundance above 0.01% across all samples were selected to construct the networks (Ma et al., 2016). The pairwise Spearman’s correlations between OTUs were calculated, with a correlation coefficient > | 0.6 | and a *P*-value < 0.01 (Benjamini and Hochberg adjusted) being considered as a valid relationship (Zhang, 2013; Lu et al., 2021; Zhang et al., 2021). In the networks, each node represents one OTU, and each edge represents a strong and significant correlation between the two nodes. Networks were visualized on the Gephi platform (Bastian et al., 2009). In addition, the global level topological features of each network, including mean node degree, clustering coefficient (CC), average path length (APL), modularity, density, and network diameter (ND), were calculated in R (Newman, 2006; Barberán et al., 2012; Ma et al., 2016; Liu et al., 2021). In addition, node-level topological features of the networks, including degree, betweenness, closeness, and eigenvector centrality, were also calculated to estimate the importance of the node. Finally, to determine the impact of SRB on the robustness of the microbial networks, we randomly deleted SRB and other taxa (the number of other taxa that were randomly deleted was the same as SRB) according to defined proportions (10 to 100%), and calculated the change of natural connectivity and the proportion of remaining nodes in R (Shi et al., 2020), every proportion was repeated 50 times.

### 2.6. Statistical analysis

Downstream analyses of 16S rRNA sequencing data were performed using the program R (v.3.4.1) (Ihaka and Gentleman, 1996). The core OTUs were identified based on their abundance-occupancy distributions (Kara and Shade, 2009). The alpha diversity metrics, including richness, evenness, and Shannon (Shannon–Wiener diversity), were calculated using the “vegan” and “picante” packages in R. For beta diversity, the principal components analysis (PCoA) based on Bray-Curtis distance and a similarity analysis (ANOSIM) were performed in the “vegan” package in R (Ihaka and Gentleman, 1996; Oksanen et al., 2015). Phylogenic diversity analyses were performed using “ses.mntd” function in the “picante” package in R. Low values of SES.MNTD indicated that taxa were closely phylogenetically clustered. Negative SES.MNTD values indicated that taxa were phylogenetically clustered more closely than expected by chance (Fan et al., 2017). Significant difference tests of diversity and node-level topological features between sediment and seawater were conducted by the Wilcoxon rank-sum test.

## 3. Results

### 3.1. Diversity and assembly of bacterial communities in sediment and seawater

**FIGURE 1:**
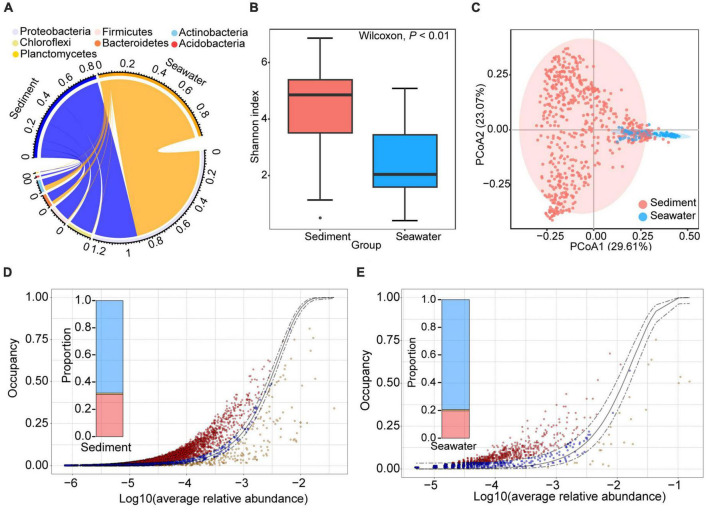
General patterns of bacterial composition, alpha- and beta-diversity in sediment and seawater. **(A)** The circus plot shows the taxonomic distribution of bacteria in sediment and seawater at the phylum level. The color of the lower semicircle corresponds to the phylum. The scale of each ribbon in lower semicircle represents the relative abundance of different phyla in sediment and seawater. If the sum of relative abundance of same phyla in sediment and seawater large than 1, the max of scale of this ribbon will large than 1. **(B)** Alpha-diversity of the bacterial community in the sediment and seawater. Shannon diversity of bacterial communities in sediment was higher than bacterial community in seawater samples. **(C)** Ordination of the bacterial community using the principal components analysis (PCoA) based on Bray-Curtis distance matrices. **(D,E)** The neutral model of bacteria community assembly in sediment **(D)** and seawater **(E)** fit to an abundance-occupancy distribution. Each point is an OTU plotted by its mean log10 relative abundance and occupancy, the solid gray line is the neutral model, and the dashed gray lines are 95% confidence intervals around the model fit. The points that fall inside the 95% model confidence are inferred to be neutrally selected (blue points). The points that fall outside the 95% model confidence are inferred to be deterministically selected (red and orange points).

After quality control, 1,073,688 and 197,931 high-quality sequences from sediment and seawater samples were clustered into 23,092 and 3,018 operational taxonomic units (OTUs) based on 97% sequence similarity, respectively. To investigate whether habitat influenced microbial community composition and diversity, we evaluated both microbial community composition and microbial diversity index. Taxonomic assignment showed that 98.67 and 68.82% of OTUs could be classified at phylum and order level, respectively. The dominant bacterial phylum in both sediment and seawater was Proteobacteria (Figure 1A). The dominant bacterial order in sediment and seawater was different. Specifically, the dominant bacterial order in sediment was *Dehalococcoidales* and *Clostridiales*. The dominant bacterial order in seawater was *Alteromonadales* and *Methylococcales* (Figure 1A and Supplementary Figure 2). Interestingly, we found that the bacterial communities in sediment were more diverse than those in seawater, and the evenness, richness, and Shannon diversity of the sediment bacterial communities were all significantly higher than those of seawater (Figure 1B and Supplementary Figure 3). Moreover, sediment and seawater samples formed distinct clusters in the ordination space, with significant differences observed at the OTUs level according to the ANOSIM test (R_*ANOSIM*_ = 0.74, *P* < 0.01) (Figure 1C).

Previous studies have demonstrated that high-diversity microbial communities experience tighter phylogenetic distribution and are governed more by deterministic processes than by stochastic processes (Danczak et al., 2018). Therefore, we further compared the differences in phylogenetic distribution and assembly processes between sediment and seawater bacterial communities. The phylogenetic clustering of microbial communities was evaluated by the mean values of the standardized effect size measure of the mean nearest taxon distance (SES.MNTD). Interestingly, we found that the SES.MNTD values of bacterial communities in both sediment and seawater were negative, indicating that the phylogenetic clustering of the bacterial community in both habitats (Supplementary Figure 3). In addition, the SES.MNTD value of the sediment bacterial community was significantly smaller than that of the seawater community (*P* < 0.01; Wilcoxon rank sum tests), suggesting that the phylogeny of the sediment bacterial community was more clustered than in seawater. This result was consistent with previous research findings, indicating that bacterial communities with high diversity in sediment experience tighter phylogenetic distribution than in seawater.

Subsequently, in order to explore whether bacterial communities in sediment were governed more by deterministic processes than by stochastic processes, we further calculated the assembly process of the bacterial communities. The result showed that the microbial communities fit the neutral community model in both habitats, as evidenced by the higher AIC of the neutral model compared to the binomial distribution model (Supplementary Figure 4). This result suggests that passive dispersal and ecological drift have an impact beyond the random sampling of the source community. Furthermore, we found that the relative contribution of stochastic processes to bacterial communities in sediment was lower than in seawater. The ratio between stochastic processes and deterministic processes in sediment (2.14) was lower than in seawater (3.85) (Figures 1D, E). Therefore, bacterial communities in sediment with high diversity experienced tight phylogenetic distribution and were governed more by deterministic processes than by stochastic processes.

### 3.2. Core microbiota and diversity of SRB in sediment and seawater

To identify the core microbiota, we used abundance-occupancy distributions to define core microbiomes. This method is an iterative exploration that quantifies the explanatory value of the core membership for beta diversity. A total of 231 and 116 OTUs were defined as the core bacterial taxa in sediment and seawater, respectively, accounting for 1.00 and 3.84% of all observed taxa. However, these taxa accounted for, on average, 46.58 and 78.68% of sequences across all samples from sediment and seawater, respectively. In order to gain insight into the potential physiological capabilities of these core bacterial communities, we performed KEGG-based PICRUSt analysis based on 16S rRNA gene profiles. Among the 231 and 116 core taxa OTUs, we found that 18 and 19 OTUs were functionally assigned to SRB in the core communities of sediment and seawater, respectively. As shown in Supplementary Table 1, both the number and mean relative abundance proportion of SRB among all OTUs were lower than the values for the core communities, indicating that SRB were enriched in the core bacterial sub-communities of sediment and seawater.

**FIGURE 2:**
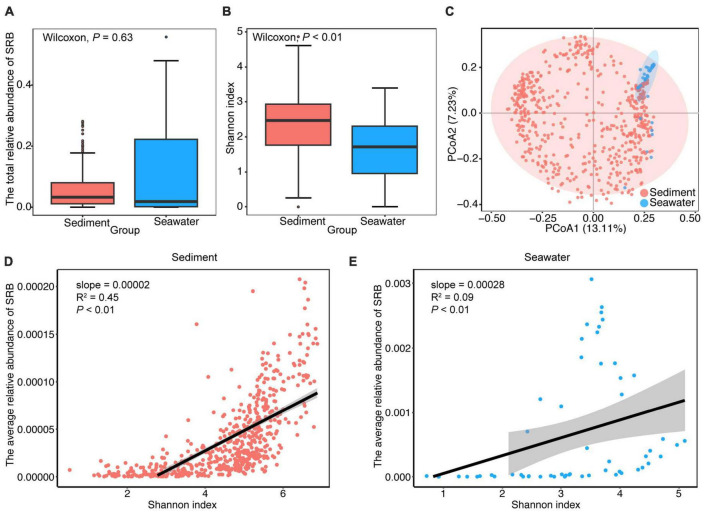
General patterns of abundance, alpha- and beta-diversity of SRB sub-community in sediment and seawater. **(A)** Comparison of the differences in total relative abundance of SRB between sediment and seawater. **(B)** Alpha-diversity of SRB sub-community in the sediment and seawater. Shannon diversity of SRB sub-community in sediment was significantly higher than seawater. **(C)** Ordination of SRB sub-community using principal components analysis (PCoA) based on Bray-Curtis distance matrices. **(D,E)** The relationship between the average relative abundance of SRB and Shannon diversity of the bacterial community in sediment and seawater. Lines denote the ordinary least-squares linear regressions.

To gain a better understanding of how the composition of the SRB sub-community varied across the different habitats, we examined the taxonomic information, abundance and diversity of the SRB sub-communities in sediment and seawater. We obtained 1,074 SRB in total. These SRB were taxonomically diverse and affiliated with 9 class and 21 order, in which 84.35% SRB were affiliated with *Deltaproteobacteria*, 39.57% SRB were affiliated with *Desulfobacterales* (Supplementary Figure 5). As shown in Figure 2, the total relative abundance of SRB was 3.34 and 1.94% in sediment and seawater, respectively. Furthermore, the significance test indicated that the total relative abundance of SRB did not vary significantly between the two habitats (*P* > 0.05; Wilcoxon rank sum tests) (Figure 2A). Regarding alpha-diversity, we found that Shannon diversity of the SRB sub-community was higher in sediment, and considerable variations were observed between sediment and seawater (*P* < 0.01; Wilcoxon rank sum tests) (Figure 2B). Although the structure of the SRB sub-community was not clearly separated at the OTUs level in the two-dimensional ordinations (Figure 2C), ANOSIM analysis indicated that the taxonomic composition of the SRB sub-community was significantly different (*R*^2^ = 0.66, *P* < 0.001) between sediment and seawater. In addition, further analysis showed significant positive correlations between the relative abundance of SRB and Shannon diversity of the community in sediment, and a similar trend was observed in seawater (Figures 2D, E).

### 3.3. Ecological roles of SRB in co-occurrence networks

**FIGURE 3:**
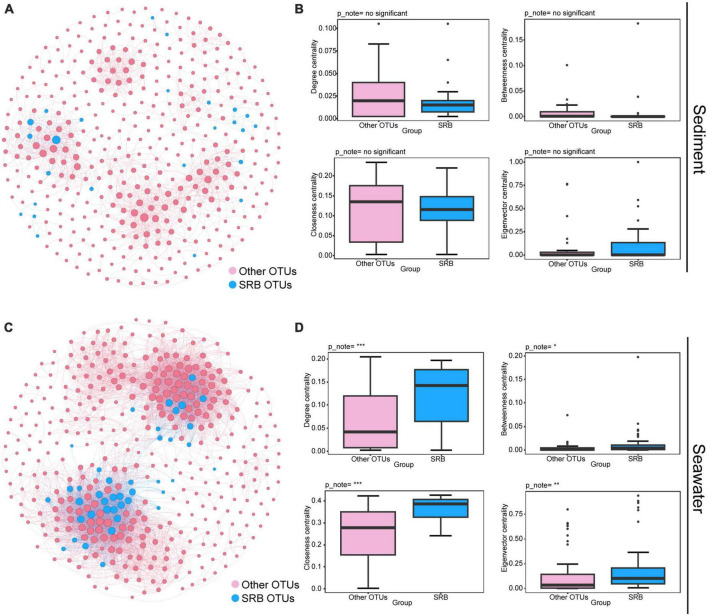
Ecological roles of the SRB in bacterial communities in sediment and seawater. Metacommunity co-occurrence networks of SRB in sediment **(A)** and seawater **(C)** based on pairwise Spearman’s correlations between OTUs. Co-occurrence networks are colored based on SRB and other OTUs. Each edge shown connection has a correlation coefficient > | 0.6| and a *P*-value < 0.01. The size of each node is proportional to the number of connections. Node-level topological features of SRB and other OTUs in sediment **(B)** and seawater **(D)**, specifically the degree, betweenness, closeness, and eigenvector centrality. All features of SRB were significantly higher than other OTUs in seawater based on Wilcoxon rank sum tests. Significance levels are as follows: *P < 0.05; **P < 0.01; ***P < 0.001.

To further investigate the ecological roles of SRB in maintaining the stability of the bacterial community, we performed the following analysis. First, we inferred a metacommunity co-occurrence network based on correlation relationships. The result showed that the correlation-based co-occurrence networks consisted of 400 nodes (OTUs) and 1,393 edges (correlations) for bacterial communities in sediment, and 402 nodes and 5,556 edges for bacterial communities in seawater. OTUs belonging to the same phylum were inclined to co-occur with one another (Supplementary Figure 6). Modularity analysis revealed seven and eight major modules (subunits with highly inter-connected nodes) in the sediment and seawater networks, respectively (Supplementary Figure 6). Many of these modules were comprised of a group of OTUs that were phylogenetically close and belonged to the same clade. This finding indicated that taxonomic relationship plays a key role in determining the network modular structure. In addition, the values of the global topological features, including density, average degree (AD), and clustering coefficient (CC) were higher in the seawater bacterial community than in sediment bacterial community (Supplementary Table 2). This result suggested that bacteria from seawater were more interconnected than those from sediment. In addition, the average path length (APL) of the sediment bacterial community was higher than of the seawater bacterial community, indicating closer relationships within bacterial communities in seawater (Supplementary Table 2). Secondly, we explored the position of SRB within the co-occurrence networks and estimated their importance to the bacterial communities by comparing the node-level topological features between SRB nodes and other nodes. As shown in Figure 3, we found that a minority of SRB in sediment had more connections to other nodes (OTUs), and node-level topological features, including degree, betweenness, closeness, and eigenvector centrality of SRB sub-communities, did not vary significantly between SRB and other nodes (Figures 3A, B). In contrast, in seawater, the majority of SRB had more connections to other nodes, and node-level topological features were both significantly higher (*P* < 0.001; Wilcoxon rank sum tests) for the SRB than for other nodes (Figures 3C, D). Altogether, these results collectively indicated that SRB possessed more connections with other nodes in seawater, in addition, the presence of SRB significantly increased network complexity in seawater. Therefore, the loss of SRB may alter network complexity and lead to further changes in network stability.

**FIGURE 4:**
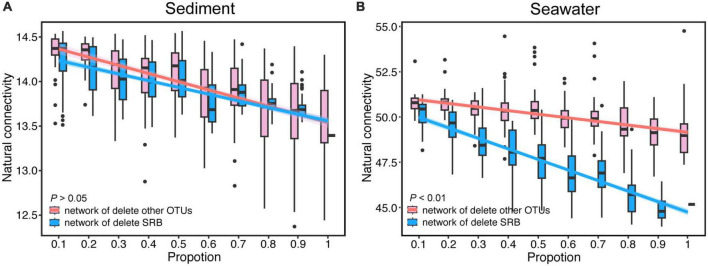
The change of natural connectivity of bacterial networks after random removal of SRB or other OTUs in sediment **(A)** and seawater **(B)** co-occurrence networks. Lines denote the ordinary least-squares linear regressions of natural connectivity.

To determine the impact of SRB on the robustness of the microbial networks, we simulated the random extinction of SRB and other nodes, and simultaneously calculated the change of the natural connectivity and the proportion of remaining nodes of network after node removal. Natural connectivity and the proportion of remaining nodes of network both reflect the robustness of network. The results showed that the natural connectivity of the bacterial networks gradually decreased with node removal in both sediment and seawater (Figure 4 and Supplementary Figure 7). Interestingly, compared to the network in which SRB nodes were randomly removed, the natural connectivity of sediment networks did not have obvious changes when other nodes were randomly removed. In contrast, the natural connectivity of seawater was dramatically decreased under SRB loss compared to the control. Additionally, the proportion of remaining nodes of bacterial networks gradually decreased with node removal in both sediment and seawater (Supplementary Figure 4). Interestingly, the proportion of remaining nodes of the sediment and seawater networks significantly decreased under SRB loss compared to the control. A comparison of the proportion of remaining nodes between the sediment and seawater networks revealed that the decrease rate of the proportion of remaining nodes in the seawater network was slower than that in the sediment network after node removal. This could be attributed to the greater interconnectivity of bacteria in seawater compared to sediment. In the seawater network, there are multiple alternative paths between nodes, and the removal of nodes did not significantly impact the connectedness of the network. Additionally, we calculated the change of average degree after node removal and discovered that the average degree in the seawater network dramatically decreased after node removal compared to the sediment network. These results indicated a strong effect of SRB on the robustness of the seawater bacterial network.

## 4. Discussion

Microorganisms form intricate webs of interactions within an ecological niche. In this study, we investigated the diversity, assembly process, and co-occurrence network of the bacterial community, while also exploring the functional taxa that have an impact on the diversity and complexity of the bacterial community. Our findings demonstrate that: (i) there are significant differences between the bacterial communities in sediment and seawater, with sediment bacterial communities possessing higher diversity and tighter phylogenetic distribution compared to seawater; (ii) stochastic processes are more pivotal in shaping the bacterial community of seawater compared to sediment. Furthermore, the network of bacterial communities in seawater is considerably more complex than that of sediment; (iii) SRB were enriched in core bacterial sub-communities, and the loss of SRB lead to a decrease in network complexity in seawater.

Microbial interactions play a central role in maintaining the diversity of microbial communities (Faust and Raes, 2012), and correlation-based network analysis has proven to be effective in exploring co-occurrence patterns and understanding microbial community structure and assembly patterns. For example, this analysis has been successfully applied to discern the direct or indirect ecological linkages among microorganisms in marine water (Beman et al., 2011; Steele et al., 2011), soil (Jiao et al., 2020), coastal sediment (Liu et al., 2020), lake (Zhang et al., 2018), and wastewater treatment plants (Ju et al., 2014).

Accordingly, in this study, we utilized network analyses to explore significant taxon co-occurrence patterns, and investigated the ecological role of the SRB in the co-occurrence networks of sediment and seawater. Our findings suggest that community composition strongly differs between sediment and seawater, with seawater possessing more complex inter-relationships among bacterial taxa than sediment. Because different groups can complement each other in a complex interaction network, the bacterial community in seawater may be more adaptable to environmental stress and possess higher functional redundancy and resiliency to environmental disturbances than sediment. Indeed, neutral model results support this notion, bacterial communities in seawater are less affected by deterministic processes than sediment. Previous studies suggested that neutral processes can modulate the occurrence frequency of species due to random fluctuations of the microbial community (Chen et al., 2017), and microbial co-occurrence associations tended to be higher when communities were primarily governed by stochastic processes (Jiao et al., 2020; Gao et al., 2021). Thus, the difference in network complexity between sediment and seawater may be due to the balance between deterministic processes and stochastic processes. In addition, the result of natural connectivity also aligns with the central ecological belief that complexity begets stability (Yuan et al., 2021), the natural connectivity of low-complexity sediment was lower than that of seawater, which had a higher community complexity.

Functional prediction results indicated that sulfate-reducing bacteria (SRB) were enriched in the core sub-communities. SRB are key participants in the ubiquitous process of sulfate reduction, which plays a critical role in the ocean ecosystem. Sulfate reduction encompasses assimilatory sulfate reduction and dissimilatory sulfate reduction, assimilatory sulfate reduction is present in all living organisms, during which sulfate is reduced to hydrogen sulfide and incorporated into cysteine and methionine, which are structural blocks for proteins and polypeptides (Schiff, 1979). Dissimilatory sulfate reduction, which is mediated by SRB, is considered the primary process for the biomineralization of organic matter in marine sediments (Jørgensen, 1982). It is estimated that 11.3 teramoles of sulfate are reduced by SRB annually. This process could account for 12 to 29% of the organic carbon being oxidized to the sea floor (Bowles et al., 2014). Therefore, dissimilatory sulfate reduction is also a crucial driver of the carbon cycle. Given the vital role of SRB in ocean ecosystems, a detailed investigation of their impact on the bacterial community is necessary to reveal their significance in the biogeochemical cycles of carbon and sulfur, and provide insight into the biological factors driving the marine sulfur cycle. In the present study, we found that the SRB sub-community in sediment had higher species richness, evenness, and diversity when compared with seawater, suggesting that the sediment SRB sub-community was more diverse and evenly distributed than seawater. Variations in species diversity are a key factor for ecological stability, and positive effects of diversity on resistance are common. Therefore, SRB in sediment and seawater may be important for the bacterial community of the ocean to resist environmental change. Additionally, in sediment, 67.09% of SRB belonged to rare taxa, for which the mean relative abundance is lower than 0.001%. While in seawater, SRB mainly belonged to abundant taxa with high abundance, and only 12.09% of SRB were rare taxa. These results indicate that SRB groups exist widely in both sediment and seawater, and that rare species contribute to the greater richness and diversity of the SRB sub-communities in sediment. Moreover, recent studies also suggest that abundant taxa have closer relationships with other taxa than rare taxa in oil-contaminated soils (Jiao et al., 2017). Abundant microbes constitute the majority of microbial biomass, and play a crucial role in carbon and nutrient cycling (Pedrós-Alió, 2012). Therefore, these sulfur cycle-related bacterial communities have considerable potential for further exploration.

Furthermore, variations in species diversity are a key factor for ecological stability, and positive effects of diversity on resistance are common. Therefore, SRB in sediment and seawater may be important for the bacterial community of the ocean to resist environmental change. In this study, we noted that seawater SRB were centrally located in their co-occurrence network, and exhibited extensive connectivity with other nodes through both direct and indirect interactions. Thus, the removal of SRB in seawater could potentially have a profound impact on the community structure, significantly reducing network complexity and ultimately leading to a decline in community stability. In order to evaluate the ecological role of SRB in the bacterial communities, we compared the node-level topological features of networks with and without SRB, and the change of natural connectivity after removing SRB or other OTUs. Our findings demonstrated that SRB in seawater possess significantly higher degree, betweenness, closeness, and eigenvector centrality than other OTUs. Recent research has shown that species with high degree centrality play a crucial role in network stability (Banerjee et al., 2018), supporting our conclusion that the presence of SRB enhances network complexity and stability in seawater. Notably, our results show that the removal of SRB significantly decreases natural connectivity compared to a network with SRB, underscoring the importance of SRB for the maintenance of network complexity and stability in seawater.

There are a few potential limitations that need to be considered when interpreting our findings. Firstly, our study relied on a topology-based approach to predict the ecological role of SRB in sediment and seawater communities. While this approach highlights the importance of SRB within the community, it does not fully capture the true inter-taxon correlations and metabolic connections between them. Therefore, further efforts are required to identify how SRB influence other taxa in the community, and to uncover the mechanisms behind the impact of SRB on community stability. For example, synthetic microbial communities and mathematical simulations could potentially be used to further explain the mechanisms behind this rule. Secondly, ecosystem stability is a multifaceted concept that is comprised of both resistance and resilience when facing disruptions (Yonatan et al., 2022). Resistance refers to the ability of the community to remain unchanged after disturbance, while resilience refers to the rate at which a community recovers to its original status after disturbance (Allison and Martiny, 2008). In our study, natural connectivity mainly refers to resistance. Due to resilience also being an essential index for evaluating community stability, further research should focus on the contribution of the SRB subcommunity to ecological resilience.

## 5. Conclusion

In summary, our study suggests that bacterial communities in seawater have high complexity, and that stochastic processes have a more crucial role in shaping seawater bacterial communities. Additionally, the diversity of both sediment and seawater bacterial communities is positively associated with the relative abundance of sulfate-reducing bacteria (SRB). Moreover, SRB in seawater play a central role and possess complex connections with other taxa in the co-occurrence network. Loss of SRB strongly decreases network complexity and stability. Our work will provide valuable insights into understanding the keystone taxa and their roles in sustaining microbial diversity and stability in ocean.

## Data availability statement

The datasets presented in this study can be found in online repositories. The names of the repository/repositories and accession number(s) can be found in this article/Supplementary material.

## Author contributions

LA, Y-CY, and H-LT collected the samples and performed the laboratory assays. C-QC and YN performed the statistical analyses. X-LW, YN, and LA designed the study, wrote and edited the manuscript. All authors read and approved the final manuscript.
