# Supplementary material for: Roles of sulfate-reducing bacteria in sustaining the diversity and stability of marine bacterial community
Source: Front Microbiol. 2023 Aug 10;14:1218828. doi: 10.3389/fmicb.2023.1218828 (PMC10448053; doi:10.3389/fmicb.2023.1218828)
Supplement: Supplementary file 1 [file Data_Sheet_1.docx]

Supplementary Material

**Roles of sulfate-reducing bacteria in sustaining the diversity and stability of marine bacterial community**

LiYun An^1^, Ying-Chun Yan^1^, Hai-Long Tian^2^, Chang-Qiao Chi^3^, Yong Nie^3*^, Xiao-Lei Wu^3,4*^

^1^ College of architecture and environment, Sichuan University, Chengdu 610065, China

^2^ College of agriculture, Henan University, Kaifeng 475000, China

^3^ College of Engineering, Peking University, Beijing 100871, China

^4^ Institute of Ocean Research, Peking University, Beijing 100871, China

**Correspondence:**
Tel: +86-010-62759047; E-mail: [nieyong@pku.edu.cn](mailto:nieyong@pku.edu.cn), [xiaolei_wu@pku.edu.cn](mailto:xiaolei_wu@pku.edu.cn)

16-digit ORCID of the authors: 0000-0002-5940-1218 (Yong Nie), 0000-0002-9897-6903 (Xiao-Lei Wu)

**Supplementary Figures and Tables**

**1 Supplementary Figures and Tables**

**1.1 Supplemental Figure:**

**
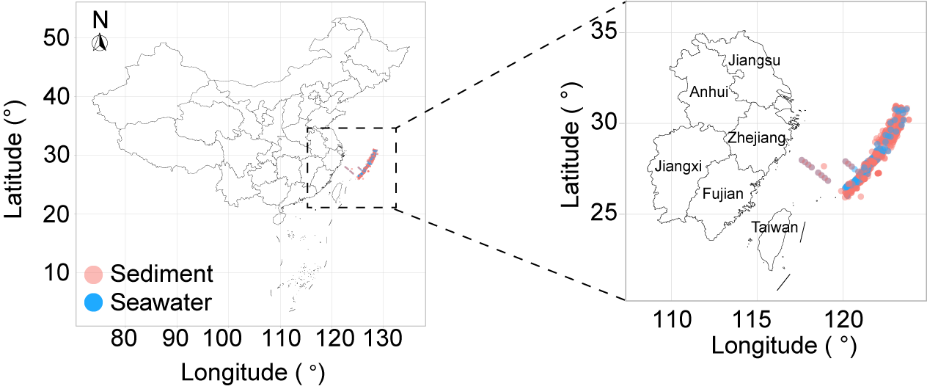
**

**Supplementary Figure 1.** The Map of sampling locations across the eastern Chinese marginal seas. A total of 594 sediment sampling sites (red dots) and 110 seawater sampling sites (blue dots) were selected ~600 km.


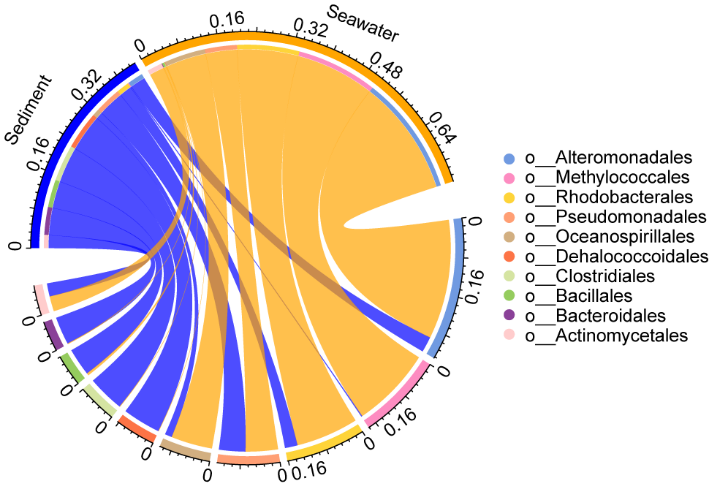


**Supplementary Figure 2.** The circus plot shows the taxonomic distribution of bacteria in sediment and seawater at the order level. The color of the lower semicircle corresponds to the order. The scale of each ribbon in lower semicircle represents the relative abundance of different order in sediment and seawater.


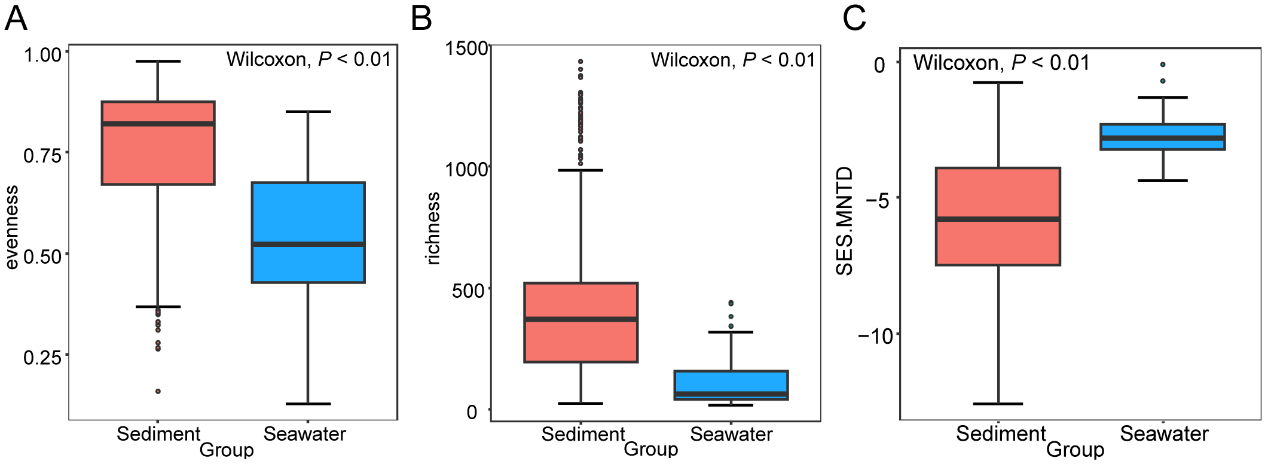


**Supplementary Figure 3.** Comparison of the differences in evenness (A), richness (B) and phylogeny diversity (C) of bacterial community between sediment and seawater.


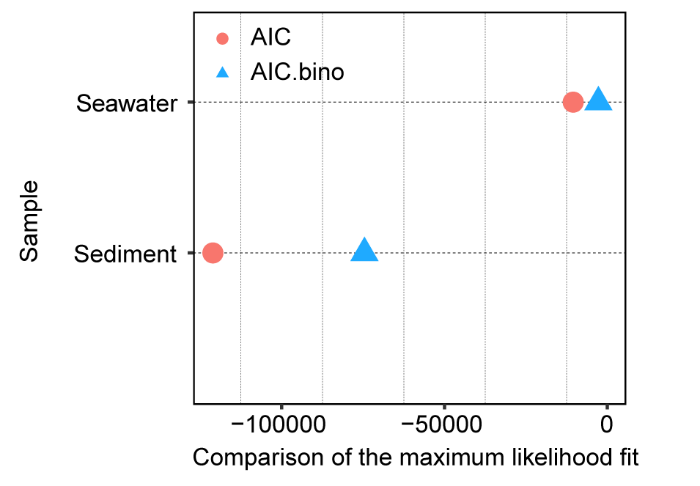


**Supplementary Figure 4.** Comparison of the maximum likelihood fit of the neutral and binomial models estimated by the Akaike information criterion for bacterial communities in sediment and seawater.


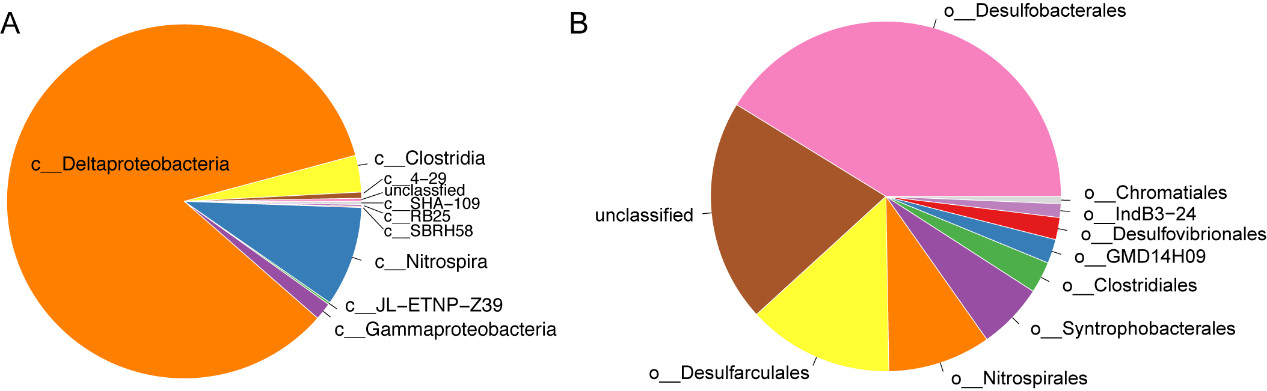


**Supplementary Figure 5.** Pie graphs showing the relative proportion and taxonomy of SRB in class level (A) and order level (B).


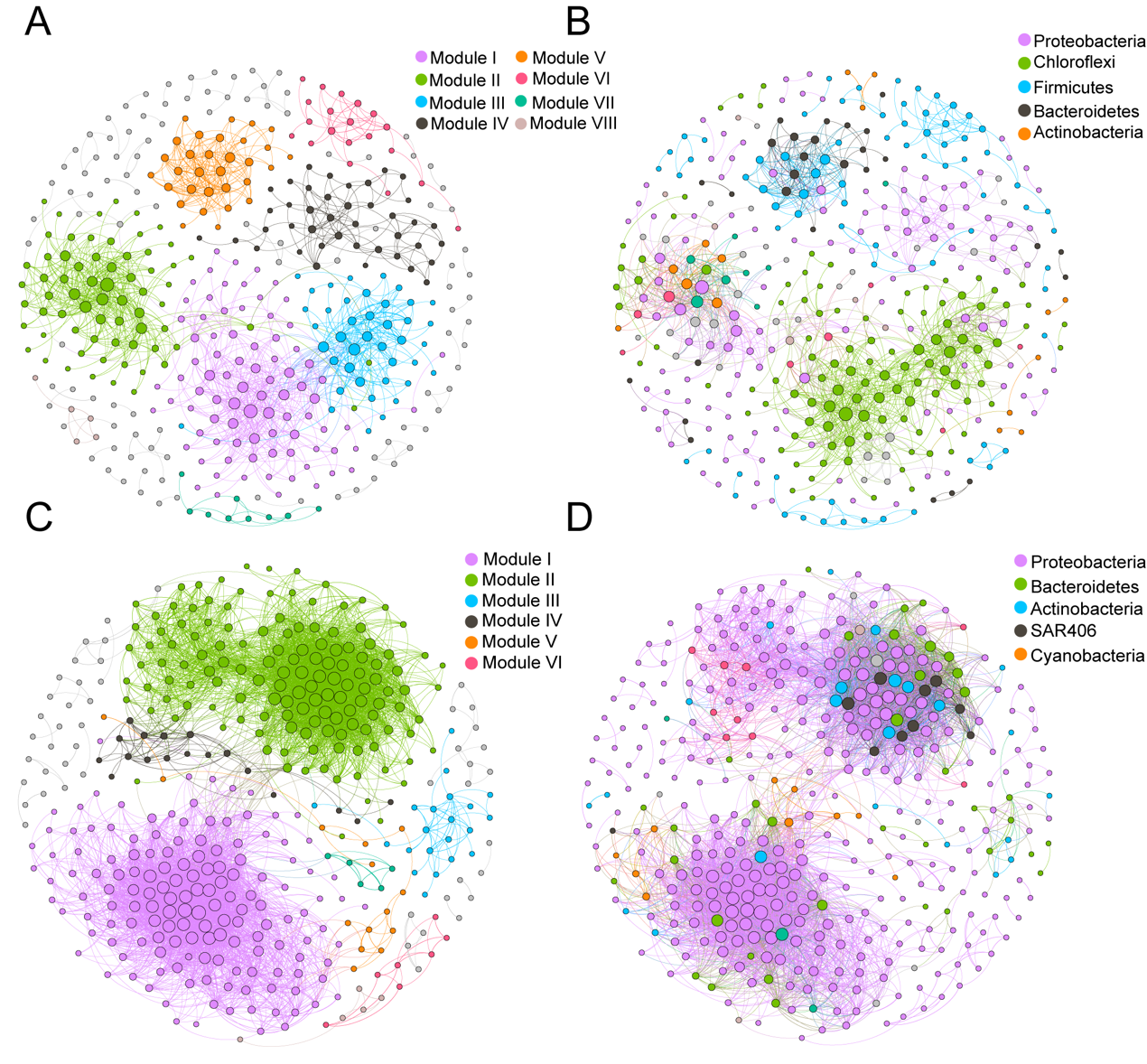


**Supplementary Figure 6.** Metacommunity co-occurrence networks based on pairwise Spearman’s correlations between OTUs. Each edge shown connection has a correlation coefficient >|0.6| and a *P* value < 0.01. The size of each node was proportional to the number of connections. The network of sediment with OTUs colored by modularity (A) and phylum (B). The network of seawater with OTUs colored by modularity (C) and phylum (D).


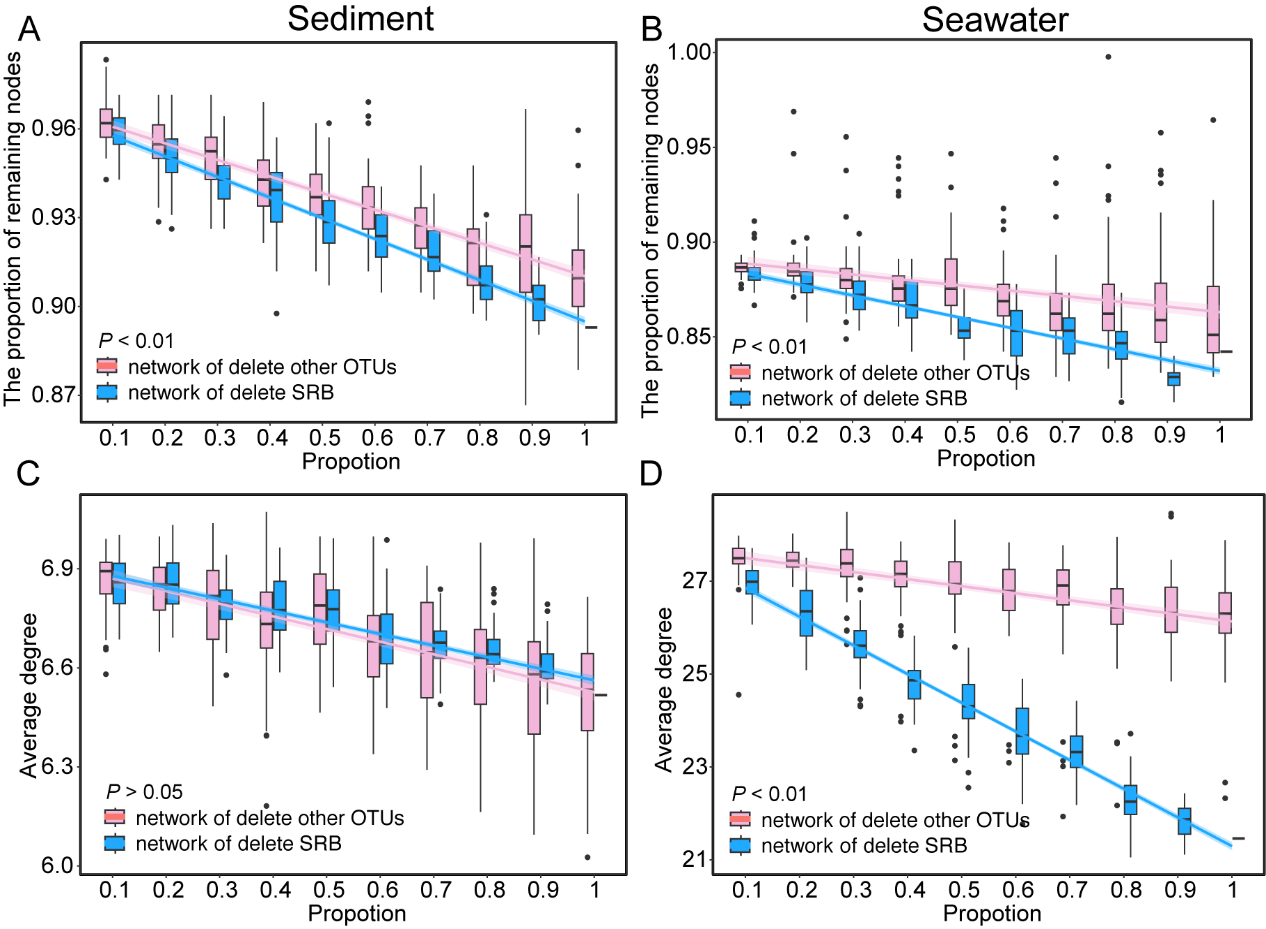


**Supplementary Figure 7.** The effect of random removal of SRB or other OTUs on the proportion of remaining nodes and average degree in sediment (A, C) and seawater (B, D) co-occurrence networks. Lines denote the ordinary least-squares linear regressions of the proportion of remaining nodes and average degree.

**1.2 Supplemental Table:**

**Supplementary Table 1.** The number and mean relative abundance proportion of SRB

|  | The proportion of number | | The proportion of average relative abundance | | |
| --- | --- | --- | --- | --- | --- |
|  | The number of SRB / the number of all OTUs | The number of core SRB/ the number of all core | | The average relative abundance of SRB / the average relative abundance of all OTUs | The average relative abundance of core SRB / the average relative abundance of all core OTUs |
| sediment | 0.059 | 0.078 | | 0.000038 | 0.0034 |
| seawater | 0.060 | 0.16 | | 0.00066 | 0.0041 |

**Supplementary Table 2.** The topological features of co-occurrence networks for different groups of bacterial taxa in sediment and seawater.

|  | Group | Density | AD | CC | APL |
| --- | --- | --- | --- | --- | --- |
| bacteria | sediment | 0.02 | 6.97 | 0.51 | 5.80 |
|  | seawater | 0.07 | 27.64 | 0.65 | 3.96 |
